# Achieving High Strength and Modulus in Bamboo/Glass Fiber Hybrid Composites Enabled by Synergistic Interfacial Design

**DOI:** 10.3390/polym18161982

**Published:** 2026-08-14

**Authors:** Jian Sun, Zhihui Li, Anqi Li, Sudong Hua, Xin Yang

**Affiliations:** College of Materials Science and Engineering, Nanjing Tech University, Nanjing 211816, China; 202361103034@njtech.edu.cn (J.S.); lizhihui@njtech.edu.cn (Z.L.); 202421031032@njtech.edu.cn (A.L.)

**Keywords:** hybrid composites, synergistic interfacial design, interfacial adhesion, mechanical properties

## Abstract

Hybrid composites combining natural and synthetic fibers offer a pathway to sustainable structural materials, yet their performance is often limited by weak interfacial bonding and mechanical mismatches between constituents. Here, we address these challenges in bamboo/glass fiber hybrid epoxy composites through a sequential alkali and silane surface modification strategy. Alkali treatment removes amorphous lignin and hemicellulose, creating a roughened, cellulose-rich surface; subsequent grafting with (3-aminopropyl) triethoxysilane introduces an amino-functionalized interphase that covalently bonds with the epoxy matrix. This combined treatment increases the tensile strength of bamboo fibers by 51.2% and the interfacial shear strength by 136.6%, reaching values comparable to those of commercial glass fibers. The resulting hybrid composite exhibits a tensile strength of 485 MPa and a flexural modulus of 30.2 GPa, corresponding to improvements of 39.4% and 98.3%, respectively, over the unmodified hybrid system. Dynamic mechanical analysis further confirms an enhanced storage modulus across a wide temperature range. This work demonstrates that rational interfacial design via sequential functionalization offers a viable route to high-performance, lightweight, and structurally stable bamboo/glass fiber hybrid composites for sustainable engineering applications, such as reusable concrete formwork.

## 1. Introduction

Against the backdrop of achieving the global 2050 carbon neutrality vision, the construction industry faces increasing pressure to reduce material consumption, lower carbon emissions, and minimize on-site energy usage. Cast-in-place concrete remains the predominant construction method worldwide. However, temporary formwork systems used to support concrete account for a significant portion of materials, labor, and waste in building construction [1]. Conventional formwork materials, such as steel, wood, and plastics, each have inherent limitations: steel formwork offers high load-bearing capacity but is heavy and energy-intensive to manufacture; wooden formwork is lightweight but suffers from low turnover rates and significant material waste; and plastic formwork provides corrosion resistance yet often lacks sufficient stiffness for demanding applications [2]. Developing lightweight, high-strength, and durable concrete formwork that overcomes these limitations therefore represents a practical and effective strategy to improve construction efficiency, enhance reusability, and reduce the environmental footprint of concrete structures.

Fiber-reinforced polymer (FRP) composites, known for their high specific strength, high specific stiffness, and excellent corrosion resistance, have been widely adopted in aerospace and transportation and are increasingly being explored for civil infrastructure applications [3,4,5,6]. Their favorable stiffness-to-weight ratio and durability make them attractive candidates for reusable or stay-in-place concrete formwork. However, despite the outstanding mechanical properties of traditional glass fibers, their non-degradability leads to persistent environmental pollution, exacerbating the global ecological burden [7] and conflicting with the sustainability goals that now guide the construction sector. This challenge has prompted academia and industry to seek more sustainable alternatives.

Natural fibers, such as bamboo fiber, are considered highly promising reinforcing materials due to their low cost, low density, renewability, and carbon-neutral characteristics [8,9,10]. However, natural-fiber-reinforced composites typically underperform as synthetic-fiber systems in terms of absolute strength, stiffness and moisture resistance. As a result, relying solely on natural fibers often fails to meet the demanding mechanical and durability requirements of concrete formwork, as well as the broader expectations of structural reliability. To address this limitation, hybrid reinforcement technology has emerged as a key research direction in materials science, as it combines synthetic and natural fibers within a single matrix to achieve complementary properties and environmental benefits [11].

The design philosophy of hybrid composites lies in leveraging the “hybridization effect” to achieve synergistic reinforcement, particularly in bending-dominated components such as concrete formwork panels [12]. Studies have shown that blending high-modulus synthetic fibers with high-specific-strength natural fibers not only reduces material density and cost but also improves the inherent brittleness and moisture sensitivity of single-natural-fiber composites through rational layup design [13,14]. Among various natural fibers, bamboo fiber is often referred to as “plant steel” due to its unique micro-gradient structure and outstanding axial tensile properties, making it an ideal candidate for hybridization with glass fiber [15,16]. This bamboo/glass fiber hybrid system thus holds promise for retaining the stiffness advantages of glass fiber while introducing the toughness and ecological benefits of bamboo fiber.

Despite this considerable potential, the full performance of glass fiber/bamboo fiber hybrid composites for lightweight structural applications such as reusable concrete formwork remains constrained by complex interfacial issues. Two main challenges stand out. First, the strong polarity and hydrophilicity of bamboo fiber surfaces create an inherent interfacial energy mismatch with hydrophobic epoxy matrices, which hinders effective bonding and limits stress transfer [17,18,19,20]. Second, the substantial disparity in physical properties between glass fibers and bamboo fibers readily induces micro-level “strain incompatibility” under loading. Without a robust interface to facilitate load transfer, this stiffness mismatch leads to stress concentration and promotes premature delamination and failure. This study utilized continuous bamboo strips as a natural reinforcing phase. Unlike short bamboo fibers or structurally damaged bamboo fibers, continuous bamboo strips retain bamboo’s inherent load-bearing framework and neatly arranged cellulose microfibrils, which help preserve its inherent structural advantages in composite materials as shows in Table 1. Sequential alkali/APTES treatment, by combining alkali-induced surface roughening and hydroxyl exposure with silane-based functionalization, further provides a practical strategy for improving interfacial performance, thereby enhancing mechanical interlock and interfacial compatibility with the epoxy matrix. In addition, surface modification and glass fiber hybridization have been widely explored as effective strategies and most previous studies have considered them separately; surface modification mainly aims to improve fiber–matrix interfacial adhesion and stress transfer, whereas hybridization with glass fibers is intended to compensate for the relatively low stiffness and strength stability of natural fibers. However, it remains unclear whether these two strategies act independently or synergistically when applied simultaneously. In particular, limited information is available on how surface-modified bamboo fibers behave in a bamboo/glass hybrid architecture and how this affects the tensile/flexural strength and modulus of the resulting composites. Previous studies on the layering sequence of bamboo and glass fibers have found that wrapping bamboo fibers around glass fibers maximizes the flexural modulus of the composite material; however, performance is still limited by the poor interfacial properties of the bamboo fiber [21]. Therefore, this study focuses on interfacial control as a key approach to improving the mechanical performance of glass fiber/bamboo fiber hybrid composites. Although laminate design, fiber orientation, and durability are also important, the laminate configuration, fiber orientation, and processing conditions were kept consistent in this work to isolate the effects of surface modification and hybridization.

Therefore, the scientific gap addressed in this work is the lack of a systematic understanding of the relationship among bamboo fiber surface modification, bamboo/glass fiber hybridization, interfacial load transfer, and macroscopic mechanical performance. This study applies a sequential alkali and silane surface modification to tailor the bamboo strips–epoxy interface in unidirectional bamboo/glass fiber hybrid composites. A multiscale characterization framework combining microscopic analyses (ATR, XPS, XRD, SEM), macroscopic mechanical testing (tensile, flexural, DMA), and numerical simulations is established to link interfacial design to overall mechanical performance. Through this approach, we elucidate how the alkali–silane treatment enhances interfacial bonding via both chemical crosslinking and mechanical interlocking, thereby mitigating the strain incompatibility between glass and bamboo fibers. The work clarifies the role of bamboo fiber surface modification under a fixed hybrid laminate configuration, excluding the influence of changes in fiber architecture or processing conditions. The findings provide a theoretical and methodological foundation for designing lightweight, high-stiffness bamboo/glass hybrid composites as sustainable panel materials for reusable concrete formwork.

## 2. Materials and Methods

### 2.1. Materials

The bamboo strips were sourced from Shengzhou City, Zhejiang Province, China. These bamboo strips are derived from five-year-old Moso bamboo, with an average length of 300 mm, a width of 4 mm, and a thickness of 0.2 mm. All fibers were harvested from the layer 2 mm beneath the bark of natural Moso bamboo grown in the same geographical location and harvested in the same year. Glass fiber plain weave fabric (EW-100A) was procured from Changzhou Xinhui Insulation Materials Co., Ltd. (Changzhou, China), with a fabric thickness of 0.1 mm ± 0.01 mm. Phenolic epoxy resin F-46 was purchased from Chuzhou Huisheng Electronic Materials Co., Ltd., Chuzhou, China. The curing agents dicyandiamide, NaOH (96%), and (3-aminopropyl) triethoxysilane (APTES, KH550, 99%) were purchased from Shanghai Aladdin Biochemical Technology Co., Ltd., Shanghai, China.

### 2.2. Surface Treatment of Bamboo Fiber and APTES Grafting

**Figure 1:**
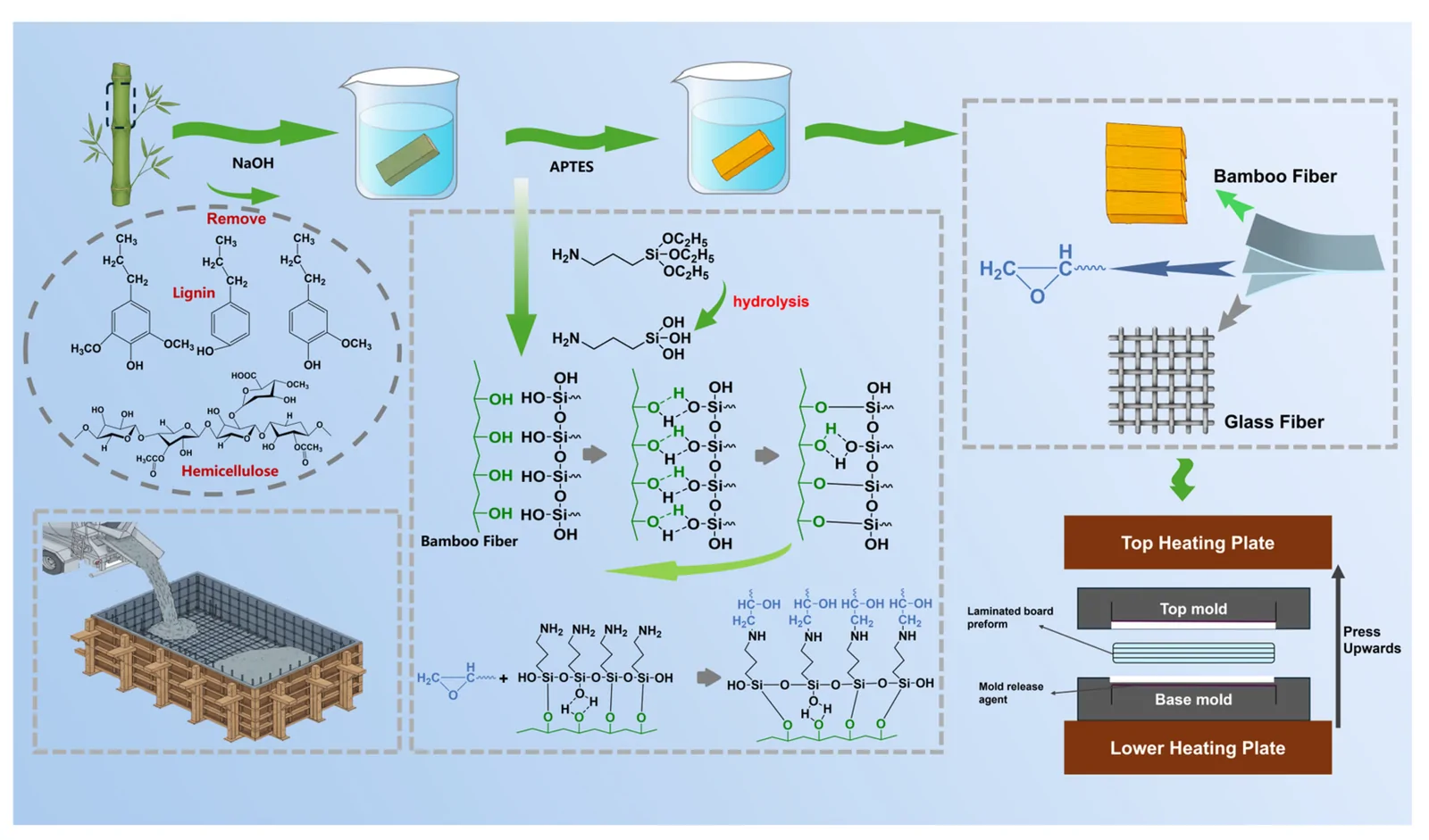
A combined interface modification strategy for bamboo to enhance the strength and modulus of bamboo–glass fiber hybrid composites.

As shown in Figure 1, the bamboo fibers were washed multiple times with deionized water and anhydrous ethanol to remove surface impurities, then dried at 80 °C for 24 h. The resulting bamboo strips were designated as “BF”. The bamboo strips were immersed in a 4% NaOH aqueous solution at a bamboo-to-solution mass ratio of 1:30 and treated at 100 °C for 12 h to partially remove lignin and hemicellulose. The treatment conditions were determined through preliminary experiments by controlling the amounts of NaOH and APTES, as shown in Table S2, and were finalized based on the surface morphology after treatment. Excessive amounts of alkali may damage the integrity of the fibers or result in uneven modification, while insufficient treatment may lead to limited surface activation. Subsequently, the bamboo fibers were boiled in deionized water for 0.5 h to generate pore structures of varying degrees on the surface, followed by soaking in deionized water for 12 h, during which the water was replaced every 2 h until the solution reached neutrality. Subsequently, the samples were dried at 80 °C for 12 h and designated as “ABF”. APTES was added to a 95% ethanol solution and stirred at room temperature for 60 min until fully dissolved and the solution became transparent. The APTES dosage was 4% of the bamboo strips’ mass. ABF was then added, and stirring continued at room temperature for 24 h. The mixture was vacuum-dried at 80 °C for 20 min. The dried fibers were activated at 120 °C for 2 h, yielding APTES-modified bamboo strips designated as “AKBF”.

### 2.3. Preparation of Hybrid Composites

A bamboo/glass fiber hybrid composite with a fiber volume fraction of 80% was prepared by layering and orienting bamboo strips bundles and glass fiber woven fabric within a mold cavity, followed by compression molding, as shown in Figure 1. Preliminary tensile tests were conducted on four types of symmetric bamboo fiber/glass fiber (BF/GF) hybrid laminates with different BF/GF ratios, as shown in Figure S1 and Table S1. The results indicate that tensile properties generally improved as the glass fiber content increased, while the bamboo fiber content decreased accordingly. Considering the balance between high natural fiber utilization and sufficient mechanical properties, BG-2 was ultimately selected as the subject for subsequent research. Subsequently, all untreated and modified composites were fabricated using the same 10-layer symmetric layup to distinguish the effects of bamboo fiber surface modification from those of the laminate structure. Therefore, the bamboo fiber-to-glass fiber ratio was set at 3:2. Next, the composite panels were placed in a vacuum bag and subjected to preforming and degassing at 70 °C to produce 300 mm × 300 mm × 2.5 mm panels. Degassing was performed for 1 h at a vacuum of 0.1 MPa to remove air voids from the pre-cured laminate. The degassed composite panels were then placed in a mold and cured for 1 h at 90 °C, followed by a 2 h post-cure at 130 °C and 6 MPa pressure. Depending on the bamboo strip treatment method, the prepared composites were named “B/G/EP,” “AB/G/EP,” and “AKB/G/EP,” respectively. In addition, the pure bamboo strip composite was designated as “BF/EP,” and the pure glass fiber composite was designated as “GF/EP.” The fiber volume fraction of the composite material was determined based on the volume ratio of fibers to resin prior to fabrication. The fiber volume fraction in the control group was the same as that in the experimental group, at 80% in both cases.

### 2.4. Characterization

Surface morphology changes before and after modification of bamboo strips were investigated using a field emission scanning electron microscope (TESCAN S8000, Tescan, Brno, Czech Republic). Powder X-ray diffraction (XRD) was employed to characterize the crystalline structure of the bamboo strips. The surface chemistry and elemental state of the bamboo strips were examined using X-ray photoelectron spectroscopy (XPS, ESCALAB 250, Thermo Fisher Scientific, Waltham, MA, USA). Thermal stability was examined using TGA (Netzsch TG 209F3, NETZSCH, Selb, Germany) within the temperature range of 35–800 °C, under a constant nitrogen flow (20 mL/min) and a heating rate of 10 °C/min. In accordance with ASTM D3039 [25] and ASTM D7264 [26], tensile specimens with dimensions of 250 mm × 15 mm × 2 mm and flexural specimens with dimensions of 100 mm × 12.5 mm × 2 mm were prepared, respectively. Flexural tests were conducted using a span-to-thickness ratio of 32:1. Five replicate tests were performed for each condition, and the results were averaged and subjected to statistical significance analysis. The tensile properties of the bamboo strips and composite materials were determined using a universal testing machine (MTS C43.504, MTS Systems Corporation, Eden Prairie, MN, USA) at a loading rate of 2 mm/min; before testing their tensile properties, the bamboo strips were vacuum-dried at 50 degrees Celsius for 12 h to eliminate errors caused by moisture absorption and degradation. For the bamboo strips, the tensile test was performed on 15 specimens, and the results were averaged; a statistical significance analysis was then performed. The IFSS was measured by a single-strip pull-out test using a universal testing machine. Bamboo strips were embedded in epoxy resin with an embedded length of 10 mm and pulled out at a crosshead speed of 2 mm/min. At least 15 valid specimens were tested for each group. Dynamic mechanical analysis of the composite material was performed using a Dynamic Thermomechanical Analyzer (DMA, NETZSCH, Selb, Germany). Measurements were conducted over a temperature range of 50 °C to 250 °C (heating rate: 5 °C/min) in single cantilever bending mode at a scanning frequency of 1 Hz.

## 3. Results and Discussion

### 3.1. The Chemical Composition and Microstructure of Bamboo Strip

The chemical structure and crystallinity evolution of natural bamboo strips (BF), delignified hemicellulose-removed bamboo strips (ABF), and silane-modified bamboo strips (AKBF) were characterized by ATR-FTIR, XPS, and XRD.

**Figure 2:**
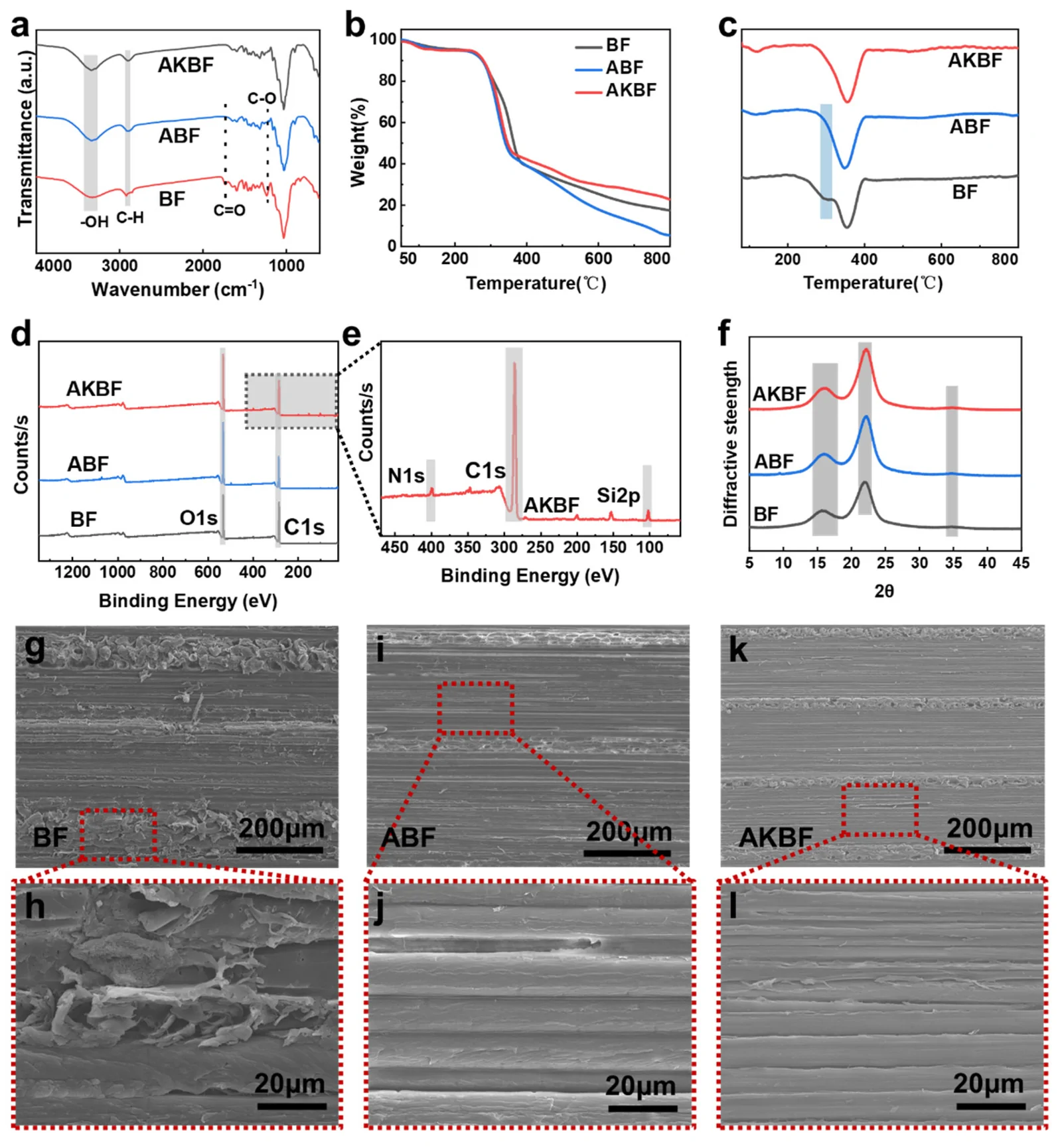
Chemical composition and microstructure of treated bamboo strips. (**a**) ATR spectra of BF, ABF, and AKBF. (**b**,**c**) Thermo-gravimetric analysis of BF, ABF, and AKBF. (**d**,**e**) XPS spectra of BF, ABF, and AKBF. (**f**) XRD spectra of BF, ABF, and AKBF. (**g**,**h**) SEM images of surface of BF. (**i**,**j**) SEM images of surface of ABF. (**k**,**l**) SEM images of surface of AKBF.

In the ATR spectra (Figure 2a), BF shows an -OH stretching vibration peak at 3340 cm^−1^ and a C-H stretching vibration peak at 2895 cm^−1^. Notably, the -OH peak intensity increases significantly after delignification, indicating greater exposure of hydroxyl groups. For ABF and AKBF, the lignin-associated peaks at 1593 and 1505 cm^−1^ weaken, while the hemicellulose peaks at 1735 and 1240 cm^−1^ disappear completely, confirming the thorough removal of hemicellulose and partial removal of lignin [27]. Throughout these treatments, the characteristic cellulose peaks (3340, 2895, 1160, 1030, and 895 cm^−1^) remain visible, suggesting that the cellulose backbone stays largely intact. The thermal degradation behavior of the modified fibers was further examined by TG and DTG (Figure 2b,c). All samples exhibit a slight mass loss of about 5% below 200 °C, corresponding to evaporation of physically adsorbed water. As the temperature rises, differences in thermal stability among the components lead to distinct degradation profiles. Untreated BF shows a clear two-step degradation in the DTG curve: a shoulder peak between 250 and 330 °C, attributed to the early pyrolysis of thermally unstable hemicellulose, followed by a main peak at 330–400 °C, corresponding to α-cellulose backbone cracking [28]. In contrast, this shoulder peak disappears completely for ABF and AKBF, leaving only a single cellulose decomposition peak in the high-temperature region. Meanwhile, lignin, owing to its complex aromatic structure, exhibits broad and slow degradation across the entire temperature range [29].

XRD analysis (Figure 2f) reveals that untreated BF exhibits the typical crystalline features of native cellulose Iβ, with distinct diffraction peaks at 2θ = 16.5°, 22.5°, and 34.6°, corresponding to the (101), (002), and (040) planes, respectively. After alkali treatment (ABF), the peak positions remain unchanged, indicating no alteration of the cellulose crystallite parameters. However, the (002) main peak becomes sharper. Quantitative calculations show that the relative crystallinity increases from 48.7% (BF) to 57.1% (ABF). This “purification” effect is attributed to the selective removal of amorphous components (e.g., hemicellulose and lignin) surrounding the microfiber surface during delignification, thereby increasing the relative content of crystalline cellulose [30,31]. Following silane modification, the XRD pattern and peak shapes show no significant changes, indicating that APTES grafting occurs via mild surface reactions between hydroxyl groups on the fiber and hydrolyzed silanol groups. This reaction does not penetrate or disrupt the tightly packed crystalline arrangement within the cellulose framework, thus preserving the intrinsic mechanical properties of the bamboo strips.

As can be seen from the XPS spectra, only peaks corresponding to C1s and O1s were observed in the broad XPS spectra of BF and ABF (Figure 2d,e), whereas the spectrum of AKBF exhibited new peaks at approximately 400 eV, 102 eV, and 150 eV, corresponding to N1s, Si2p, and Si2s, respectively. As shown in the atomic ratios presented in Table S3, the O/C ratio increased from 0.353 to 0.638 after alkali treatment, indicating that the hydroxyl-rich fiber surface was exposed, thereby providing active sites for subsequent silane modification. For AKBF, the presence of Si and N and the corresponding Si/C and N/Si ratios further confirm that the APTES-derived silane structure has been incorporated into the fiber surface.

As clearly shown in Figure 2g–l, the chemical treatment process not only cleans the bamboo strip surface but also significantly reshapes its microscopic topological structure, which is a critical factor in determining the interfacial bonding mechanisms of the resulting composites.

The surface of untreated bamboo strips (BF, Figure 2g,h) exhibits a rough texture characterized by extensive coverage of amorphous impurities. As illustrated in the schematic, this layer is primarily composed of wax, pectin, and lignin, which collectively form a loose, shell-like barrier that tightly encapsulates the internal cellulose skeleton. This structure obscures the fibers’ inherent texture and creates a natural, weak boundary layer between the strips and the polymer matrix. Such a barrier not only physically impedes effective resin impregnation but also serves as a preferential site for crack initiation, leading to premature interfacial failure when the composite is subjected to mechanical stress.

In contrast, treatment with NaOH (ABF, Figure 2i,j) leads to the complete disruption of this impurity barrier. The alkaline solution dissolves binding hemicellulose and partial lignin, causing the previously bonded elementary fibers to dissociate. This process reveals distinct, deep longitudinal grooves on the strip surface. This is not merely a cleaning process; it significantly increases the specific surface area of the fiber by creating a rich micro-texture. These newly formed grooves and cavities provide ideal physical anchor points for the resin matrix, thereby enhancing mechanical interlocking at the interface [32].

Following subsequent treatment with APTES (AKBF, Figure 2k,l), the strip surface undergoes a distinct morphological transformation. Unlike the sharp, exposed grooves of the ABF, the APTES-treated strip surface exhibits a unique restorative compactness, appearing to be enveloped by a uniform, continuous film. This results in a smoother and more even surface morphology compared to the ABF. These subtle changes in microstructure, together with the presence of silicon and nitrogen previously observed by XPS, confirm the successful self-assembly of APTES on the bamboo strip’s surface. The resulting polysiloxane network not only fills micro-cracks and repairs surface defects left by the alkali treatment but also grafts a layer of molecular bridges bearing active amino groups onto the surface. This establishes the material foundation for transitioning the interfacial adhesion mechanism from purely physical interlocking to a more robust chemical bonding regime [33].

### 3.2. Mechanical Properties of Bamboo Strip and Interfacial Shear Strength

**Figure 3:**
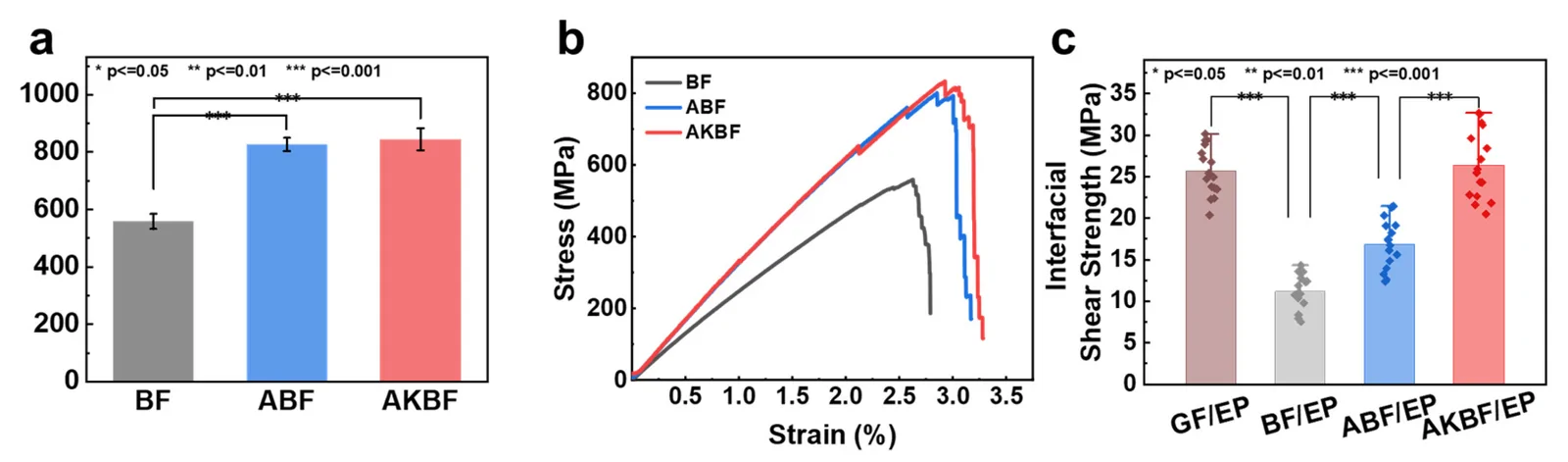
(**a**) Tensile strength of the BF, ABF and AKBF. (**b**) Tensile stress–strain curves for BF, ABF and AKBF. (**c**) Interfacial shear strength between fiber and resin.

Figure 3a shows the effect of chemical treatment on the intrinsic mechanical properties of individual bamboo strips. The tensile strength of ABF increased from 558.38 MPa for BF to 826.62 MPa, and further increased to 844.25 MPa for AKBF, corresponding to an overall improvement of 51.2%. This strengthening effect is primarily attributed to the structural densification of bamboo strips following delignification. The process selectively removes hemicellulose and amorphous lignin, which act as structural defect sources, thereby reducing the microcrack density. At the same time, it promotes a more compact and ordered axial alignment of the load-bearing cellulose microfibrils, as corroborated by the increased crystallinity observed in the XRD analysis [34].

Figure 3b presents the tensile stress–strain curves of bamboo strips, which exhibit multi-stage damage during fracture. This behavior arises because the bamboo strips contain minute defects randomly distributed throughout the strips. The strip itself can be viewed as a natural composite in which bamboo microfibers reinforce a lignin matrix. Owing to the low strength and uneven distribution of lignin, stress concentration easily occurs under loading, causing cracks to initiate in the outer fibers and then propagate randomly inward until final failure.

Notably, after silane modification, the tensile strength of AKBF remains comparable to that of ABF, with no statistically significant difference. This observation reveals the “non-destructive” nature of APTES modification. As a confined surface treatment, the silanization reaction constructs only a nanoscale functional coating on the outermost layer of the fiber, without penetrating or disrupting the crystalline integrity of the cellulose backbone [35]. Consequently, this dual-treatment strategy endows the fiber surface with high chemical reactivity while fully preserving its excellent intrinsic mechanical properties, thereby laying a solid foundation for enhancing the performance of the resulting composites.

Interfacial shear strength (IFSS) is a key parameter for evaluating the efficiency of stress transfer between fibers and the matrix in composite materials. The interfacial shear strength between the fibers and the resin matrix was determined through interfacial pull-out tests of bamboo strips and epoxy resin. As shown in Figure 3c, surface modification has a decisive influence on interfacial bonding performance, exhibiting a clear stepwise enhancement. The untreated BF exhibits the lowest IFSS value of 11.15 MPa, reflecting the inherent weakness of the pristine interface. This poor bonding performance primarily stems from the accumulation of impurities on the surface of the native bamboo strips. These chemically inert substances reduce surface energy and hinder effective wetting by the epoxy resin, forming a weak boundary layer at the interface. Consequently, the interfacial bond relies solely on weak van der Waals forces and limited physical friction, making it difficult to maintain structural integrity under high loads.

After delignification, the IFSS increases significantly to 15.86 MPa, representing a 42.2% improvement over the untreated state (11.15 MPa). This enhancement is mainly attributed to the activation of mechanical interlocking mechanisms. Following the removal of surface impurities by the alkaline solution, the cellulose microfibril framework becomes exposed and roughened, as confirmed by the microstructural analysis. This topographical reconstruction substantially increases the specific surface area, allowing the liquid resin to infiltrate surface micropores and, upon curing, form physical anchors that markedly improve pull-out resistance. In addition, alkali treatment removes surface impurities and exposes more hydroxyl groups, which can enhance hydrogen bonding interactions between the fibers and the epoxy resin.

Remarkably, after silane functionalization, the IFSS of AKBF further increases to 26.39 MPa, which is 136.6% higher than that of the untreated bamboo strip. This breakthrough result unequivocally demonstrates the crucial role of the APTES coupling agent in interfacial reinforcement. Specifically, the silanol groups of the APTES molecules condense with surface hydroxyl groups to form Si-O-C bonds, while the terminal active amino groups penetrate the resin matrix and participate in the curing crosslinking reaction. This synergistic combination of strong chemical bonding and physical anchoring fundamentally alters the stress transfer pattern at the interface, enabling natural fibers to achieve bonding performance comparable to that of synthetic fibers.

### 3.3. Dynamic Thermomechanical Properties of Composite Materials

**Figure 4:**
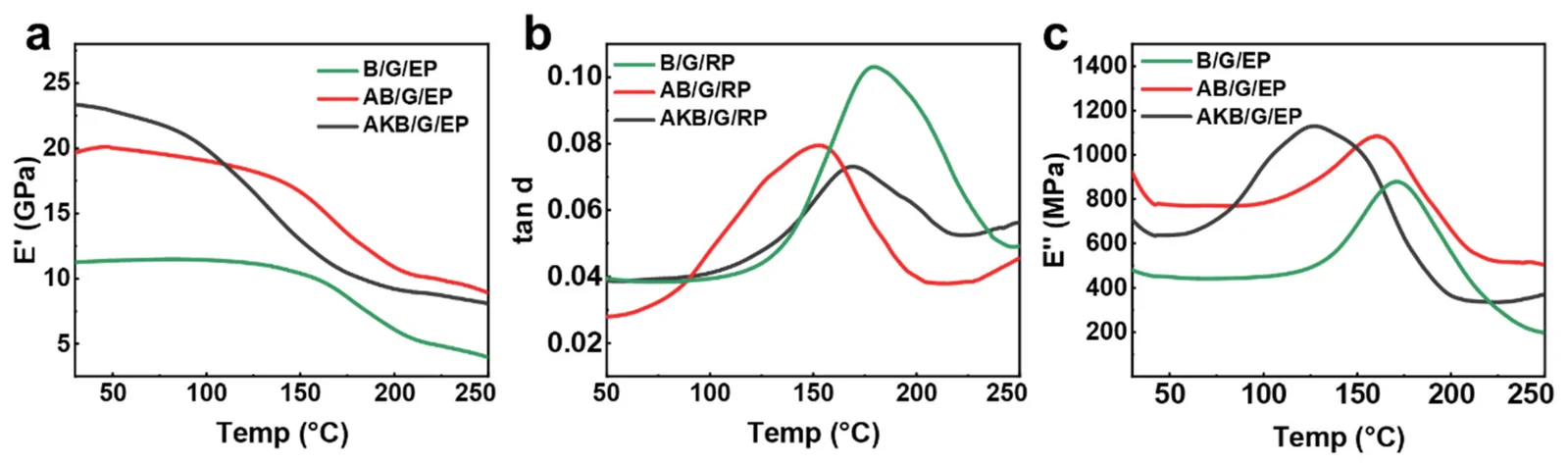
Dynamic thermal mechanical analysis of hybrid composites. (**a**) Storage modulus of composites. (**b**) Loss modulus of composites. (**c**) Tan δ of composites.

Figure 4a shows the temperature-dependent storage modulus (E′) of the three composites. In the glassy region (50 °C), the modified composites exhibit substantially higher E′. Specifically, AB/G/EP and AKB/G/EP reach 22,851 MPa and 23,288 MPa, respectively, which are about 101% and 105% higher than that of B/G/EP. As temperature rises above Tg, E′ decreases for all samples, but the decay rate differs. At 50 °C above the respective Tg, the E′ of B/G/EP drops to 4670 MPa, whereas AB/G/EP and AKB/G/EP retain 9138 MPa and 6079 MPa, respectively. Notably, AB/G/EP at 50 °C above its Tg retains approximately 1.96 times the E′ of B/G/EP. These results indicate that interfacial modification enhances both low-temperature stiffness and post-softening load transfer [36,37].

The E′-T curves suggest that alkali treatment transforms bamboo strips from a filler into an effective reinforcement by removing surface lignin/hemicellulose, increasing roughness, and introducing polar groups. The additional silane treatment further introduces a chemically crosslinked layer at the interface, which maintains the highest initial stiffness and superior high-temperature performance across the entire temperature range.

Figure 4b presents the tan δ curves. All composites show a single α-relaxation peak, but the peak positions differ. The Tg (tan δ peak) of B/G/EP is 180 °C. Alkali treatment lowers the Tg of AB/G/EP to 152 °C, the Tg of the composite decreased by 28 °C, indicating that alkali treatment alone reduced the thermal margin of the composite and the decrease in Tg is attributed to the removal of rigid lignin/hemicellulose and the exposure of hydrophilic hydroxyl groups and possible changes in the local crosslink density of the epoxy near the treated bamboo surface [38]. From an application perspective, although alkali treatment reduced the Tg of AB/G/EP, the measured Tg remained above the expected temperature range encountered by formwork during ordinary concrete casting. However, long-term durability under coupled thermal, humid, and alkaline environments should be further evaluated before practical application. In addition, the combined alkali silane treatment raises the Tg of AKB/G/EP to 206 °C, the silane coupling agent forms Si-O-fiber bonds and its organic groups participate in the epoxy network, creating a rigid interfacial layer that restricts segmental motion and delays α-relaxation to higher temperatures. Additionally, the tan δ peak heights of the modified samples are reduced, indicating suppressed interfacial slip and viscoelastic dissipation [39]. Thus, both modifications, especially the combined treatment, are interface-enhanced instead of viscosity-enhanced, consistent with the IFSS results.

Figure 4c shows the loss modulus (E″) curves. B/G/EP exhibits an E″ peak at 172 °C (878 MPa). For AB/G/EP, the peak shifts to 127 °C and increases to 1133 MPa. For AKB/G/EP, the peak returns to ~170 °C and further increases to 1327 MPa. The loss modulus peak of AB/G/EP shifted to a lower temperature and showed a higher intensity, indicating enhanced molecular and interfacial energy dissipation. This increased dissipation may be beneficial for damping or impact-energy absorption; however, it also reflects increased chain mobility at lower temperatures. In contrast, the combined treatment shifts the primary dissipation process to higher temperatures while maintaining a high dissipation capability at increased stiffness. Notably, in the rubbery region at 50 °C above Tg, both AB/G/EP and AKB/G/EP retain a high storage modulus (E′) and a considerable loss modulus (E″), which is beneficial for their application in thermo-mechanically coupled environments.

In summary, alkali treatment improves wetting and mechanical interlocking by removing inert components, increasing roughness, and introducing polar groups, thereby transforming bamboo strips into effective reinforcements. However, it also removes some of the rigid amorphous phases, and the increased water absorption resulting from the exposure of hydroxyl groups leads to a decrease in Tg. Therefore, alkali treatment alone may be detrimental to the material’s high-temperature stability. Combined alkali–silane treatment simultaneously enhances E′ across the entire temperature range, reduces tan δ peak height, and shifts both Tg and the E″ peak to higher temperatures, thus maintaining high stiffness and dissipation capability at elevated temperatures. These DMA results are fully consistent with the observed fiber morphology and IFSS trends, confirming that the combined alkali pretreatment and silane coupling strategy effectively achieves ordered interfacial construction in bamboo fiber/glass fiber hybrid composites.

### 3.4. Mechanical Properties of Composites

**Figure 5:**
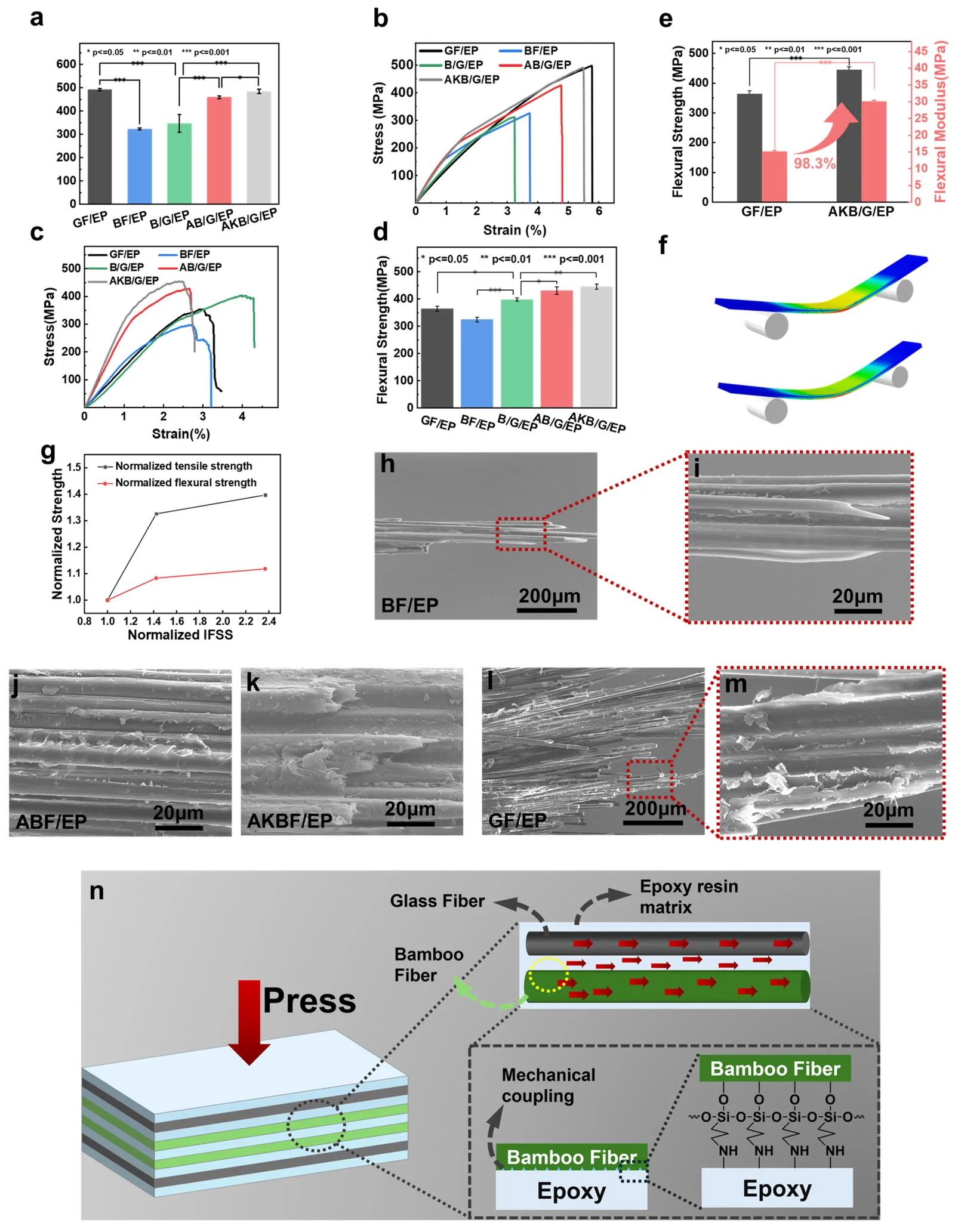
Analysis of mechanical properties and reinforcement mechanism of hybrid composites with combined interfacial engineering optimization. (**a**–**d**) Mechanical properties of hybrid composites. (**e**,**f**) Flexural strength and flexural modulus of composites, together with simulation validation. (**g**) Semi-quantitative correlation between normalized IFSS and normalized composite strength. (**h**–**m**) SEM images of fracture surface morphologies of composites made from different fibers. (**n**) Load-transfer mechanisms between bamboo strips and the matrix under bending loads.

Figure 5a–d presents the mechanical properties of the composites before and after bamboo strip modification. Interfacial modification clearly enhances the macroscopic mechanical performance of the composites. Under tensile loading, the unmodified B/G/EP composite exhibits a strength of only 346.5 MPa, which is considerably lower than that of the pure glass fiber reference group. Since glass fibers possess higher intrinsic strength and stiffness than bamboo fibers, the mechanical properties of B/G/EP were expected to fall between those of BF/EP and GF/EP. The experimental results showed the same trend, indicating that the untreated hybrid composite mainly reflects the load-sharing behavior of the two reinforcements. The lower performance of B/G/EP compared with GF/EP is attributed to the lower intrinsic properties and weaker interfacial bonding of untreated bamboo fibers. As shown in Figure 2g,h, the untreated bamboo strip surface, rich in pectin and wax, creates distinct physical gaps between the fiber and the epoxy matrix. These microscopic voids cannot efficiently transfer shear stress under load; instead, they act as stress concentration sites, promoting microcrack initiation and rapid propagation at stress levels far below the fiber’s ultimate strength, thereby leading to premature material failure. In contrast, the tensile strength of the dual-modified AKB/G/EP composite increases to 484.2 MPa, corresponding to a 39.4% improvement. This significant enhancement may result from the synergistic combination of physical anchoring and chemical bonding. First, alkali treatment removes non-cellulosic impurities from the fiber surface, exposing a rough cellulose skeleton that increases the specific surface area and provides ideal mechanical interlocking points for the resin. Second, the silane coupling agent (APTES) hydrolyzes to form silanol groups, which condense with hydroxyl groups on the bamboo strip surface, while its organic functional groups form covalent bonds with the epoxy resin matrix. This “molecular bridge” structure creates a rigid transition layer between the fibers and the matrix, substantially increasing the interfacial shear strength (IFSS) [40,41]. Previously, IFSS results showed that the IFSS of AKBF/EP increased by 136.6% compared to BF/EP. This confirms that effective interfacial bonding can significantly improve the tensile properties of composite materials. However, it is worth noting that while the tensile properties of AKB/G/EP have improved compared to those of AB/G/EP, the improvement is relatively limited. Based on the previously observed patterns of improvement in the tensile properties of bamboo strips, it can be inferred that, under tensile loading, the intrinsic tensile properties of the reinforcing bamboo strips have a more significant impact on the tensile properties of the composite material than the interfacial properties do.

Bending tests involve more complex stress states, including tension on the tensile side, compression on the compressive side, and interlaminar shear near the neutral axis. Although the glass fiber composite exhibits extremely high tensile strength, its flexural strength is only 364.3 MPa. The addition of bamboo strips increases the flexural strength to 398 MPa. After interfacial modification of the bamboo strips, the flexural strength of AKB/G/EP further increases to 445.1 MPa. The stress–strain curves reveal that, after interfacial optimization, the flexural modulus and stiffness of the hybrid fiber composite significantly exceed those of the single-fiber composites. This improvement stems not only from the enhanced interfacial tensile strength described above but also from the densification of the fiber–matrix interface and its resistance to interlaminar peeling. On the compression side of the bend, natural fibers are highly susceptible to micro-buckling, which can lead to structural collapse. However, alkali treatment removes the lignin and hemicellulose that fill the spaces between cellulose microfibrils, allowing the microfibrils to rearrange and pack more tightly under surface tension. This significantly increases the radial compressive modulus of the fiber bundle and improves the material’s resistance to local buckling [42].

Dual modification provides a stronger interfacial bond between the bamboo strips and the resin. This is expected to allow stress to be transmitted uniformly between the fiber layers, thereby preventing the interlaminar delamination commonly observed during bending. This enhanced interlaminar adhesion ensures the thickness integrity of the hybrid structure, enabling the outer glass fiber layers to provide effective stress shielding for the inner bamboo strip core [43]. Furthermore, compared to pure glass fiber composites, this hybrid dual-modified composite not only significantly improves flexural strength but also increases the flexural modulus by approximately 98.3%. A finite element model was established in Abaqus to simulate the notchless bending response of a composite laminate consisting of two different composite materials. The composite plies were represented by shell elements, and the interfaces between adjacent plies were described using a cohesive zone model. The laminate layup and geometric dimensions were consistent with those used in the experimental tests. A displacement-controlled three-point bending configuration was applied, with the specimen supported at both ends and loaded at the mid-span. Using this simulation method, a qualitative analysis of the stress distribution in composite laminates under bending loads was conducted. As shown in the lower part of Figure 5f, the AKB/G/EP composite exhibited a more uniform stress distribution, further confirming the ultrahigh modulus imparted by the hybrid fibers.

The IFSS and composite strengths were normalized using BF/G/EP as the reference to evaluate their semi-quantitative relationship (Tables S4 and S5). As shown in Figure 5g, both tensile and flexural strengths increased with increasing normalized IFSS, indicating that enhanced interfacial adhesion facilitated stress transfer and contributed to improved mechanical performance. Specifically, when the normalized IFSS increased to 1.422 and 2.367, the normalized tensile strength increased to 1.326 and 1.397, and the normalized flexural strength increased to 1.083 and 1.118, respectively. However, the strength enhancement was not proportional to the IFSS increase, suggesting that the macroscopic properties were also affected by fiber integrity, hybrid structure, matrix behavior, and defects. Therefore, this relationship is discussed as a semi-quantitative trend rather than a strict predictive correlation. To evaluate the applicability of the developed hybrid composite, its flexural properties were compared with those of previously reported fiber-reinforced wood formwork panels. Previous studies have shown that fiber reinforcement is an effective strategy for improving the mechanical properties of bio-based formwork panels. Liu [44] reported a carbon fiber fabric-reinforced poplar/eucalyptus plywood for construction formwork, which exhibited a maximum longitudinal modulus of elasticity (MOE) of 15.55 GPa. In this study, the strength and modulus of the bamboo/glass fiber hybrid composite were also enhanced following interfacial modification. Although direct comparisons are limited due to differences in the types of reinforcing materials, matrices, panel structures, and testing standards, these results suggest that bamboo/glass fiber hybrid composites may be a promising lightweight panel material.

To further analyze the relationship between interfacial strength and the mechanical properties of composite materials at the microscopic level, Figure 5h–m shows scanning electron microscopy images of the fracture morphologies. In the unmodified BF/EP composite (Figure 5h), fibers are pulled out relatively intact, exhibiting smooth surfaces. At higher magnification (Figure 5i), almost no residual resin is observed on the fiber surfaces. After delignification (Figure 5j), the fracture surface becomes noticeably rougher, with clear resin residue on the fiber surfaces and in the inter-fiber voids. This is attributed to the opening of voids between bamboo microfibrils after delignification, which allows the resin to penetrate more readily into the bamboo strip and form a denser physical interlock. In addition, the removal of surface impurities further facilitates resin impregnation. The APTES-treated AKB/G/EP composite (Figure 5k) exhibits a distinctly different fracture morphology, with more resin masses adhering to the fiber surfaces in a fragmented state. The fiber fracture surfaces are enveloped by resin, indicating that cracks propagate perpendicular to the fiber direction during composite failure. The high IFSS prevents energy dissipation through fiber pull-out, forcing dissipation through brittle fracture instead. As illustrated in Figure 5n, when a load is applied, it is uniformly distributed between the fibers and the resin through strong covalent bonds. The fracture morphology of the glass fibers (Figure 5l,m) shows fiber clusters, with a few scattered pulled-out fibers exhibiting minimal surface resin residue. However, most fibers remain clustered together, still held by the resin. This indicates that, during composite failure, a small number of glass fibers debond from the resin, allowing cracks to initiate at the debonded regions. These cracks then propagate transversely through the glass fiber bundles. Subsequently, the failure of fiber bundles that remain strongly adhered to the resin leads to the overall failure of the composite.

## 4. Conclusions

This study developed a bamboo/glass fiber hybrid epoxy composite. The main challenges are the incompatibility between hydrophilic bamboo strips and the hydrophobic epoxy matrix, and the strain mismatch between the two reinforcements. To address these issues, a sequential alkali and silane surface modification was applied to tailor the bamboo–epoxy interface. Alkali treatment removed amorphous lignin and hemicellulose, creating a rough, cellulose-rich surface with higher crystallinity and exposed hydroxyl groups. This improved mechanical interlocking and reduced interfacial energy mismatch.

The subsequent silane surface grafting treatment effectively improved the interfacial shear strength between the bamboo strips and the epoxy resin, further enhancing load transfer efficiency and mitigating strain incompatibility. The combined treatment increased the tensile strength of bamboo strips by 51.2% and the interfacial shear strength by 136.6%, which is a significant improvement compared to before processing. Relative to the unmodified system, the resulting hybrid composite showed a tensile strength of 484.2 MPa (a 39.4% improvement) and a flexural modulus of 30.1 GPa (a 98% improvement). Dynamic mechanical analysis also revealed a higher storage modulus over a wide temperature range. These findings indicate that rational interface design through sequential functionalization is a viable approach to overcoming the key challenges faced by bamboo–glass hybrid composites. This method facilitates the development of lightweight, high-strength, and structurally stable materials suitable for sustainable engineering applications, offering a potential high-performance, environmentally friendly alternative to traditional concrete formwork. However, this study evaluated only the interfacial and mechanical properties at the material level. Before being put into practical use, the material still requires further structural design verification, full-scale panel testing, cyclic loading tests, moisture and alkali resistance tests, and long-term durability assessments.

## Figures and Tables

**Table 1:** Mechanical properties of the bamboo–glass hybrid and other hybrid composites.

Fiber Composition	Method	Tensile Strength (MPa)	Flexural Strength (MPa)	References
Bamboo/Glass Fiber	Hand-Lay Up + ComPression Molding	485.0	445.1	This Work
Bamboo/Glass Fiber	Vacuum Infusion	87.2	99.7	[21]
Banana/Glass Fiber	Hand-Lay Up	80.9	145.4	[22]
False Banana/Glass Fiber	Hand-Lay up	9.3–35.7	54.7–222.1	[23]
Sisal/Glass Fiber	Hand-Lay Up + ComPression Molding	48.0	159.0	[24]

## Data Availability

The raw data supporting the conclusions of this article will be made available by the authors upon request.

## Supplementary Materials

The following supporting information can be downloaded at: https://www.mdpi.com/article/10.3390/polym18161982/s1, Figure S1: Tensile properties of hybrid composites with different layup configurations. (a) Tensile stress-strain curves for hybrid composites. (b) Tensile strength of hybrid composites; Figure S2: SEM images of (a) ABF-1; (b) ABF-4; (c) ABF-7; (d) AKBF-1; (e) AKBF-4; and (f) AKBF-7; Table S1: Fiber ratios and layup methods for BF and GF; Table S2: Compositions of different bamboo fiber samples; Table S3: Surface elemental composition and atomic ratios of BF, ABF, and AKBF determined by XPS; Table S4: Normalized IFSS of composites for semi-quantitative correlation analysis; Table S5: Normalized tensile strength, and flexural strength of composites for semi-quantitative correlation analysis.
