# Supplementary material for: Achieving High Strength and Modulus in Bamboo/Glass Fiber Hybrid Composites Enabled by Synergistic Interfacial Design
Source: Polymers (Basel). 2026 Aug 14;18(16):1982. doi: 10.3390/polym18161982 (PMC13516411; doi:10.3390/polym18161982)
Supplement: Supplementary file 1 [file polymers-18-01982-s001.zip › polymers-4436008-supplementary.pdf]

Supporting Information

Achieving High Strength and Modulus in Bamboo/Glass Fiber Hybrid Composites Enabled by Synergistic Interfacial Design

Jian Sun, Zhihui Li, Anqi Li, Sudong Hua * and Xin Yang *

College of Materials Science and Engineering, Nanjing Tech University, Nanjing 211816, China; 202361103034@njtech.edu.cn (J.S.); lizhihui@njtech.edu.cn (Z.L.); 202421031032@njtech.edu.cn (A.L.)
* Correspondence: huasudong@njtech.edu.cn (S.H.); xin12.25@hotmail.com (X.Y.)

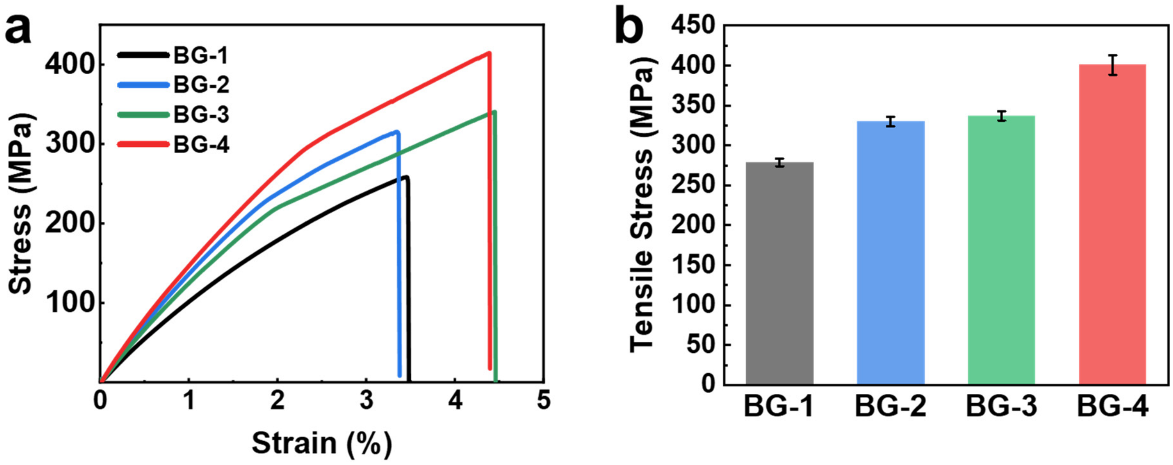

Figure. S1. Tensile properties of hybrid composites with different layup configurations. (a) Tensile stress-strain curves for hybrid composites. (b) Tensile strength of hybrid composites.

All composites with the four fiber ratios were fabricated using a symmetric layup. For the BG-1 laminate, bamboo fiber (BF) plies were used as the outermost layers on both surfaces, whereas glass fiber (GF) plies served as the outermost layers for the remaining laminates. As shown in Figure S1, the tensile properties generally improved with increasing GF content. In Figure S1a, BG-3 (BF:GF = 2:3) exhibits a markedly higher elongation at break than BG-2; however, the overall tensile properties of these two laminates are not significantly different. Considering the trade-off between maximizing the natural-fiber content and maintaining

adequate mechanical performance, all modified groups were fabricated using the BG-2 layup configuration. To ensure specimen comparability and minimize structural effects, all laminates were prepared as 10-ply symmetric stacks with the sequence [GF/BF/BF/GF/BF/BF/GF/BF/BF/GF].

Table S1. Fiber ratios and layup methods for BF and GF.

Samples	BF/GF fiber volume ratio	Layering Methods for BF and GF
BG-1	BF:GF = 4:1	BF/BF/GF/BF/BF/BF/BF/GF/BF/BF
BG-2	BF:GF = 3:2	GF/BF/BF/GF/BF/BF/GF/BF/BF/GF
BG-3	BF:GF = 2:3	GF/GF/BF/BF/GF/GF/BF/BF/GF/GF
BG-4	BF:GF = 1:4	GF/GF/BF/GF/GF/GF/GF/BF/GF/GF

Prior to selecting the treatment parameters for the main study, preliminary screening experiments were conducted using different concentrations of alkali and silane. For the alkali treatment, NaOH solutions with concentrations of 1 wt.%, 4 wt.%, and 7 wt.% were compared; for the subsequent silane modification, APTES solutions with concentrations of 1 wt.%, 4 wt.%, and 7 wt.% were evaluated. Table S2 summarizes the corresponding masses of NaOH and APTES used to prepare each treatment solution.

The purpose of these preliminary experiments was to determine treatment conditions that would effectively modify the surface of the bamboo strips while avoiding excessive surface damage. The treated bamboo strips were examined using a scanning electron microscope (SEM), and surface morphology was used as the primary criterion for qualitative screening.

As shown in Figures S2(a–c), these surface impurities are gradually removed as the concentration of the alkaline treatment solution increases. When the sodium hydroxide

concentration is too low (see Figure S2a), the lignin and hemicellulose coating the surface of the cellulose macrofibrils cannot be effectively removed. Conversely, an excessively high NaOH concentration (see Figure S2c) leads to alkaline degradation of the cellulose macrofibrils themselves, causing the primary cell walls to dissolve and the microfibrils to peel off from the surface, thereby compromising the structural integrity of the cellulose macrofibrils. As shown in Figures S2d–f, a rough, film-like coating forms on the surface after APTES grafting. An APTES concentration that is too low can easily result in insufficient silane grafting, while an excess of APTES can induce self-coagulation, leading to the accumulation of silanol crystals on the surface of the bamboo strips.

Table S2 Compositions of different bamboo fiber samples

Sample	Bamboo strips(g)	NaOH(g)	APTES(g)
BF	30	0	0
ABF-1	30	9	0
ABF-4	30	36	0
ABF-7	30	63	0
AKBF-1	30	36	0.3
AKBF-4	30	36	1.2
AKBF-7	30	36	2.1

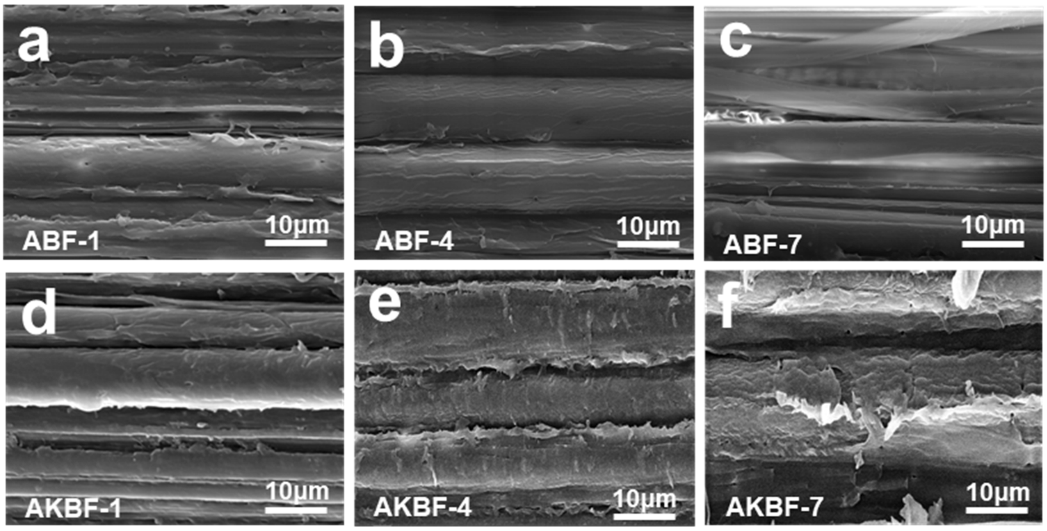

Figure. S2. SEM images of (a) ABF-1; (b) ABF-4; (c) ABF-7; (d) AKBF-1; (e) AKBF-4; and (f) AKBF-7.

Table S3. Surface elemental composition and atomic ratios of BF, ABF, and AKBF determined by XPS.

Sample	Elemental Content (at%)				Ratio of Elements		
	C	O	Si	N	O/C	Si/C	N/Si
BF	73.91	26.09	ND	ND	0.353	--	--
ABF	61.04	38.96	ND	ND	0.638	--	--
AKBF	64.09	30.94	2.74	2.23	0.483	0.043	0.814

To establish a semi-quantitative relationship between interfacial adhesion and macroscopic mechanical performance, the interfacial shear strength (IFSS), tensile strength, and flexural strength were normalized using the untreated BF/G/EP composite as the reference. This normalization allows different properties with different units and magnitudes to be compared on the same relative scale.

For a given property *X*, the normalized value was calculated as:

$$X_{norm,i} = \frac{\bar{X}_i}{\bar{X}_{ref}}$$

where $X_{norm,i}$ is the normalized value of sample *i*, $\bar{X}_i$is the mean experimental value of sample *I*, and $\bar{X}_{ref}$ is the mean value of the untreated B/G/EP reference composite. In this study, *X* represents IFSS, tensile strength, or flexural strength. Therefore, the normalized values were calculated as:

$$\tau_{norm,i} = \frac{\bar{\tau}_i}{\bar{\tau}_{ref}}$$

$$\sigma_{t,norm,i} = \frac{\bar{\sigma}_{t,i}}{\bar{\sigma}_{t,ref}}$$

$$\sigma_{f,norm,i} = \frac{\bar{\sigma}_{f,i}}{\bar{\sigma}_{f,ref}}$$

where τ, σ_t, and σ_f represent IFSS, tensile strength, and flexural strength, respectively. The normalized value of the untreated B/G/EP composite was defined as 1.000 for each property.

Table S4. Normalized IFSS of composites for semi-quantitative correlation analysis.

Samples	IFSS (MPa)	Normalized IFSS
BF/EP	11.15±2.833	1
ABF/EP	15.86±3.608	1.422
AKBF/EP	26.39±4.596	2.367

Table S5. Normalized tensile strength, and flexural strength of composites for semi-quantitative correlation analysis.

Samples	Tensile strength (MPa)	Normalized Tensile strength	Flexural strength (MPa)	Normalized Flexural strength
B/G/EP	491.9±5.06	1	398.0±6.01	1
AB/G/EP	323.3±4.48	1.326	431.1±13.80	1.083
AKB/G/EP	346.5±7.97	1.397	445.1±9.65	1.118
